# Combination of immune checkpoint inhibitors with radiation therapy in cancer: A hammer breaking the wall of resistance

**DOI:** 10.3389/fonc.2022.1035884

**Published:** 2022-12-05

**Authors:** Veronika Voronova, Anastasia Vislobokova, Kerim Mutig, Mikhail Samsonov, Kirill Peskov, Marina Sekacheva, Maria Materenchuk, Natalya Bunyatyan, Svetlana Lebedeva

**Affiliations:** ^1^ Department of Pharmacological Modeling, M&S Decisions LLC, Moscow, Russia; ^2^ Department of Pharmacology, Institute of Pharmacy, I.M. Sechenov First Moscow State Medical University, Moscow, Russia; ^3^ MID3 Research Center, I.M. Sechenov First Moscow State Medical University, Moscow, Russia; ^4^ Artificial Intelligence Research Center, STU Sirius, Sochi, Russia; ^5^ World-Class Research Center “Digital biodesign and personalized healthcare”, I.M. Sechenov First Moscow State Medical University, Moscow, Russia; ^6^ Institute of Professional Education, I.M. Sechenov First Moscow State Medical University, Moscow, Russia; ^7^ Federal State Budgetary Institution “Scientific Centre for Expert Evaluation of Medicinal Products” of the Ministry of Health of the Russian Federation, Moscow, Russia

**Keywords:** clinical trials, combination therapy, immune checkpoint inhibitors, immuno-oncology, radiotherapy

## Abstract

Immuno-oncology is an emerging field in the treatment of oncological diseases, that is based on recruitment of the host immune system to attack the tumor. Radiation exposure may help to unlock the potential of the immune activating agents by enhancing the antigen release and presentation, attraction of immunocompetent cells to the inflammation site, and eliminating the tumor cells by phagocytosis, thereby leading to an overall enhancement of the immune response. Numerous preclinical studies in mouse models of glioma, murine melanoma, extracranial cancer, or colorectal cancer have contributed to determination of the optimal radiotherapy fractionation, as well as the radio- and immunotherapy sequencing strategies for maximizing the antitumor activity of the treatment regimen. At the same time, efficacy of combined radio- and immunotherapy has been actively investigated in clinical trials of metastatic melanoma, non-small-cell lung cancer and renal cell carcinoma. The present review summarizes the current advancements and challenges related to the aforementioned treatment approach.

## Principles of cancer immunotherapy

Recruiting the patient’s immune system for cancer treatment was proposed by the American surgeon William Coley back in 1891 (1). Using bacterial cultures and their metabolic products he developed a vaccine to treat patients with inoperable tumors. Despite the positive results of his research, this treatment strategy did not received an approval and was soon replaced by chemotherapy (CT) and radiation therapy (RT).

**Figure 1:**
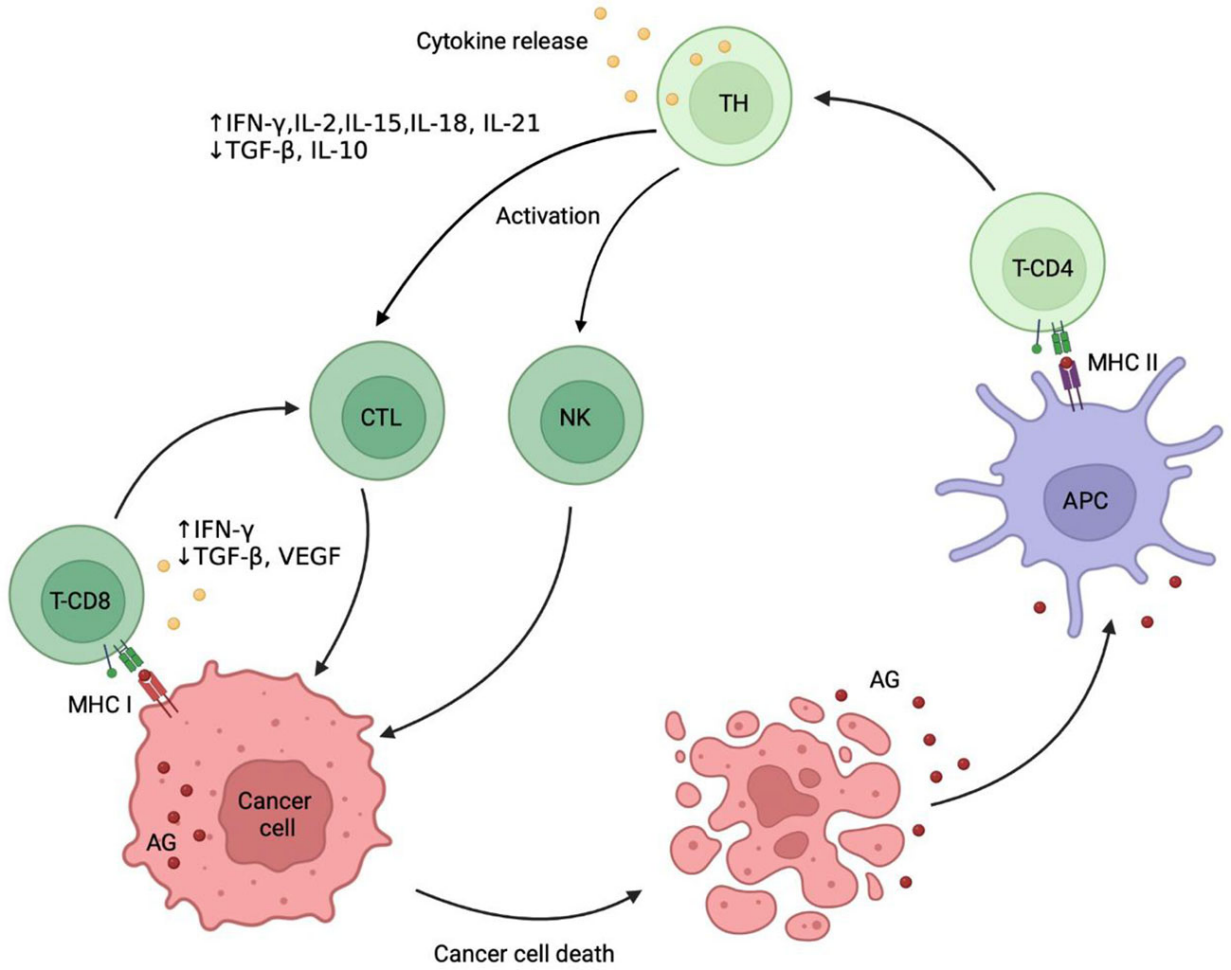
Cancer immunity cycle. Immunogenic death of tumor cells leads to release of tumor-specific antigens (AG) that are captured by antigen presenting cells (APC) and after binding to major histocompatibility complex (MHC) class II are presented on their surface. The receptors CD4 dock to an MHC class II, activate the naive T helper (TH) cells followed by clonal selection of antigen-specific T cells, their proliferation, migration into the tumor site and differentiation into cytotoxic T-lymphocytes (CTL). CTL express receptors CD8, which dock to MHC class I. Finally, CTL recognize tumor-specific antigens and kill the tumor cells, which produces a further release of tumor antigens into the surrounding space and potentiates the immune response. Natural killer (NK) cells recognize tumor cells without the involvement of the MHC class I, making the response mediated very quick. AG, tumor-associated antigens; MHC I, major histocompatibility complex class I; MHC II, major histocompatibility complex class II; TH, T helper cell; NK, natural killer cell.

The progress in understanding the molecular mechanisms of immunity in the late 20th century, including the role of various cell populations and cytokines in the activation, maintenance and downregulation of the immune response, revitalized the idea of using the host immune system against the tumor cells (2–4). According to the current concept, proposed by D. Chen and I. Mellman (2013) (5), specific antigens released from tumor cells during stress-induced immunogenic cell death (ICD) stimulate clonal expansion of the tumor-specific T-lymphocyte subsets. Next, dendritic cells (DCs) present the antigens on major histocompatibility complex (MHC) class I and MHC class II molecules to T cells. The activated T cells attack the tumor cells and enhance the antitumor immune response. The effects of ICD stimulate the recruitment of T cells and their ability to recognize the tumor cells (6) (**Figure 1**).

However, practical observations show that the activation of the immune response is not always sufficient for tumor rejection, which can lead to natural selection of cancer cells, resistant to the immune attack due to multiple immunosuppressive mechanisms. This phenomenon has been termed immunoediting (3, 7). The resistant tumor cell populations recruit various mechanisms to create a tumor microenvironment (TME), which not only reduces the immune response but also stimulates tumor growth. Therefore, one of the main challenges of the current oncotherapy is to shift TME from the immunosuppressive to the immunoreactive state, which requires establishing and implementation of new therapeutic strategies. Immune tolerance was shown to be driven by multiple TME components including immunosuppressive cell populations such as regulatory T (Treg) cells and myeloid derived suppressive cells, various cytokines, soluble factors, enzymes and metabolites (e.g. arginase, adenosine, TGF-β) (8).

Immune checkpoint (IC) proteins were shown to play an important role in the immunosuppression and have been increasingly recognized as an important target for anticancer drug development. In non-pathological conditions IC proteins prevent hyperactivation of the immune system and are crucial for immune tolerance. Cancer cells, however, use these proteins to limit the specific antitumor immune response, thus protecting themselves from the T cell attack. The programmed death 1 (PD-1), its ligand PD-L1, and cytotoxic T-lymphocyte-associated antigen 4 (CTLA-4) are the best characterized ICs.

PD-1 is a receptor expressed on the surface of T- and B-lymphocytes, macrophages and myeloid cells serving to suppress the autoimmunity *via* specific modulation of apoptotic signaling mechanisms in distinct populations of immunocompetent cells (9, 10). PD-1 is expressed both in cancer and antigen presenting cells (APC). Binding of PD-L1 to PD-1 activates the Src homology-2-containing protein tyrosine phosphatase 2 (SHP2), which inhibits the phosphatidylinositol-3-kinase (PI3k)/protein kinase B (PKB/AKT) signaling pathway by dephosphorylation of the phosphatase and tensin homolog (PTEN), thereby suppressing the downstream molecular pathways including AKT, mitogen-activated protein kinase (MAPK), extracellular signal-regulated kinase (ERK) and others (11–15). Deactivation of these cascades leads to a decrease in lymphocytes proliferative activity and effector functions (16, 17).

PD-L1 up-regulation in tumors occurs either by constitutive oncogenic signaling *via* AKT or signal transducer and activator of transcription 3 (STAT3), a mechanism termed intrinsic immune resistance, or by interferon gamma (IFN-γ) released from activated T cells or natural killer (NK) cells (adaptive resistance) (18). High PD-1 and PD-L1 expression was detected in several tumor cell lines, such as serous ovarian carcinoma (10) and breast cancer (19), although the prognostic value of PD-1 or PD-L1 expression is currently unclear (10, 20). Moreover, conventional cancer therapies such as radio- or chemotherapy have been associated with activation of the PD-1 signaling in tumor cells conferring a certain resistance against these treatments (21–23).

CTLA-4 represents another potential therapeutic target among ICs. CTLA-4 appears on the surface of T cells after their interactions with APC and prevents the T cells activation by competing with CD28 for its ligands, CD80 and CD86. CTLA-4 has a greater affinity for CD28-activating ligands than CD28 itself. The resulting CD28 antagonism suppresses the PI3k/AKT cascade (24–27). In contrast to the PD-1 signaling, CTLA-4 likely acts *via* the protein phosphatase 2A (PP2A) to decrease the abundance of phosphorylated AKT (15, 28, 29). CTLA-4 is overexpressed under pathophysiological conditions in the tumor environment, which substantially reduces the availability of CD80 and CD86 for the CD28-pathway and limits the proper activation of T-lymphocytes at early maturation stages (14).

CTLA-4 and PD-1 act as negative regulators of T cells function at different stages of the immune response: CTLA-4 regulates the proliferation of T cells in the early stages, primarily in the lymph nodes, whereas PD-1 plays an important role in the regulation of previously activated T cells, predominantly in peripheral tissues (9, 16).

## Clinical application of immune checkpoint inhibitors

Monoclonal antibodies to PD-1, PD-L1 or CTLA-4 are emerging agents in cancer therapy due to their potential to block the excessive IC activity and restore the antitumor immune response. To date, several preparations of monoclonal antibodies affecting various ICs have been developed and approved for a wide range of cancer treatments (**Table 1**) and multiple IC inhibitors (ICI) are currently at the stage of preclinical or clinical trials (30).

**Table 1:** Approved ICIs according to FDA labels.

Drug name, company	Approval year	Target	Indications
**IPILIMUMAB** **(YERVOY)** BRISTOL MYERS SQUIBB	2011	CTLA-4	Melanoma Renal cell carcinoma Colorectal cancer Hepatocellular carcinoma Non-small cell lung cancer
**PEMBROLIZUMAB** **(KEYTRUDA)** MERCK SHARP DOHME	2014	PD-1	Melanoma Non-small cell lung cancer Small cell lung cancer Head and neck squamous cell cancer Classical Hodgkin Lymphoma Urothelial carcinoma Microsatellite Instability-High or Mismatch Repair Deficient Cancer Microsatellite Instability-High or Mismatch Repair Deficient Colorectal Cancer Gastric Cancer Esophageal Cancer Cervical Cancer Hepatocellular Carcinoma Merkel Cell Carcinoma Renal Cell Carcinoma Endometrial Carcinoma Tumor Mutational Burden-High Cancer Cutaneous Squamous Cell Carcinoma
**NIVOLUMAB** **(OPDIVO)** BRISTOL MYERS SQUIBB	2015	PD-1	Melanoma Non-small cell lung cancer
**ATEZOLIZUMAB** **(TECENTRIQ)** GENENTECH INC	2016	PD-L1	Urothelial Carcinoma Non-Small Cell Lung Cancer Triple-Negative Breast Cancer Small Cell Lung Cancer Hepatocellular Carcinoma
**AVELUMAB** **(BAVENCIO)** EMD SERONO INC	2017	PD-L1	Merkel Cell Carcinoma Urothelial Carcinoma Renal Cell Carcinoma
**DURVALUMAB** **(IMFINZI)** ASTRAZENECA UK LTD	2017	PD-L1	Urothelial carcinoma Non-small cell lung cancer Extensive-stage small cell lung cancer
**CEMIPLIMAB-RWLC** **(LIBTAYO)** REGENERON PHARMACEUTICALS	2018	PD-1	Сutaneous squamous cell carcinoma

The first ICI ipilimumab, which is an antibody to CTLA-4, was approved by FDA for treatment of metastatic inoperable melanoma in 2011 (31). The results demonstrated an improved long-term survival over 5 years (more than 18% of patients receiving the treatment), which led to a broad clinical and scientific resonance and stimulated studies of other ICIs.

Nivolumab and pembrolizumab were further medications approved by FDA for treatment of metastatic inoperable melanoma in 2014 and small cell lung carcinoma in 2015. Atezolizumab (PD-L1-specific monoclonal antibody) was approved for the therapy of the urothelial carcinoma in 2016. Further PD-L1-specific antibodies, avelumab and durvalumab, received the FDA support for the treatment of urothelial carcinoma in 2017. In addition, a PD-1-specific monoclonal antibody, cemiplimab, has been approved by FDA for the therapy of squamous cell skin cancer in 2018 (31). Preliminary and early results of these studies indicate that ICIs may bear a broad therapeutic potential for tumors of various histological composition including treatment of advanced metastatic or non-metastatic cancer stages.

## Overcoming resistance to immune checkpoint inhibitors

Although, the ICIs have demonstrated clinical efficacy in patients with various types of cancer (see **Table 1**), the inconsistency of patients’ responses and a relatively high percentage of non-responsive patients remain a major limitation for this therapy. According to various sources, up to 80% of patients with previously treated and advanced non-small-cell lung cancer (NSCLC), recurrent squamous-cell carcinoma of the head and neck, melanoma, as well as relapsed or refractory Hodgkin’s lymphoma showed no adequate response to ICI treatment (32–41).

The response to ICIs has been shown to depend on various factors including the presence of certain T cell and APC subpopulations, immunosuppressive cytokine responses, levels of antigenic molecule inhibition in malignant cells, recruitment of immunoregulatory cells of the myeloid and lymphoid series to the neoplasm area, and dysfunction of DCs (10).

Multiple mechanisms of the tumor resistance to the ICI have been identified. These mechanisms can be classified into the primary mechanisms due to insufficient tumor recognition by the immune system, the adaptive mechanisms related to the immune response downregulation, and the acquired mechanisms driven by the immunoediting of the tumor (42).

Managing the resistance to immune therapy depends on the kind of therapeutic intervention and should be, as much as possible, personalized in each patient. The resistance to ICI can be determined by the absence of tumor antigenic proteins (primary resistance) or development of mechanisms decreasing antigen presentation and enabling immune evasion (secondary resistance). Multiple primary and adaptive tumor-intrinsic mechanisms include signaling through the MAPK pathway and/or loss of PTEN expression, which enhances the PI3K and WNT/β-catenin signaling pathways partially *via* suppression of the IFNγ (42). In addition, tumors of patients non-responding to the anti-PD-1 therapy showed signs of epithelial-mesenchymal transformation, which may further promote the tumor survival (43). The composition of molecular and cellular tumor microenvironment seems to be critical to the anti-tumor immune response.

The strategies to overcome resistance to the immunotherapies are aimed at selection of patient populations which are likely to respond to the treatment or using combination strategies to target diverse immunosuppressive pathways.

Assessment of PD-L1 and PD-1 as biomarkers of primary and metastatic tumors is often required for making clinical decisions on use of treatment strategies targeting ICs (44). Clinical studies of PD-1/PD-L1 blocking antibodies with patient stratification according to the PD-L1 expression at the tumor site showed a higher overall and relapse free survival, as well as a greater number of responses to therapy in patient groups with a higher biomarker level (45). At the same time, the use of one factor in stratification of patients for the treatment order may not be sufficient considering the complex TME structure. Therefore, various combined biomarker strategies, including tumor genome profiling, assessment of the T-cell repertoire, and studies of TME are currently being tested to develop multivariate predictive models for assessing the likelihood of successful treatment outcomes (46–49).

Combination strategies to combat resistance include coadministration of several immunotherapeutic agents with various mechanisms of action, as well as combinations with chemo-, radio- or targeted therapies (50). Combined administration of CTLA-4 and PD-1/PD-L1 blocking antibodies has been extensively investigated as an option to improve their therapeutic efficacy. To date, the only approved treatment combination consists of ipilimumab and nivolumab. This combination was approbated in 2015 for metastatic melanoma treatment and a 60% response was demonstrated (31, 51, 52). At a minimum follow-up of 60 months, the median overall survival was more than 60.0 months in the nivolumab-plus-ipilimumab group and 36.9 months in the nivolumab group, as compared with 19.9 months in the ipilimumab group (53). Combined nivolumab and ipilimumab therapy has been approved for various indications (54) and demonstrated sustained overall survival benefit in renal cell carcinoma (RCC) (54, 55) and in NSCLC (56). In 2020, the combination of ipilimumab and nivolumab received a further FDA approvement for the treatment of adults with malignant pleural mesothelioma (MPM) that cannot be removed by surgery (57). Other immunotherapy combinations are currently being investigated in clinical settings including coadministration of the registered ICIs with vaccines, other ICIs, adoptive cell transfer or agents, targeting various immunosuppressive TME components (58, 59). Generally, the choice of drug combinations for an optimal treatment is a complex task requiring careful consideration of their individual and synergistic therapeutic vs. side effects. It should be also noted that optimization of combined treatments is challenging and includes not only rationale selection of therapeutic modalities, but also the optimization of dosing regimens for each of them as well as their sequence.

One of the potential effective approaches to treat patients with cancer is a combination of CT and ICI. Some cytotoxic chemotherapeutic drugs such as anthracycline and oxaliplatin could induce ICD and stimulate antitumor immune response (60). Nowadays, CT combined with α-PD-1/PD-L1 has become a standard option for some cancer patient categories. The efficacy and safety of such combinations has been confirmed by a large number of clinical trials. Patients with NSCLC receiving pembrolizumab combined with standard CT (carboplatin and pemetrexed) had a higher response rate and longer progression-free survival, as well as overall survival than patients receiving standard CT only. As a consequence, pembrolizumab plus CT has been approved by the FDA as the first-line treatment for advanced non-squamous NSCLC, regardless of the PD-L1 level. Subsequently, the indication of pembrolizumab plus CT was expanded to the advanced triple-negative breast cancer (TNBC), esophageal cancer, and gastroesophageal junction cancer (GEJC). Apart from α-PD-1 the α-PD-L1-based chemoimmunotherapy attracts an intensive attention too, especially the drug atezolizumab. Based on the results of IMpower150, which is a pioneer clinical trial assessing the efficacy of atezolizumab plus angiogenesis inhibitor and CT in patients with advanced non-squamous NSCLC, the FDA approved atezolizumab plus bevacizumab, paclitaxel, and carboplatin as the first-line treatment for advanced non squamous NSCLC. Later, the FDA approved atezolizumab plus CT for TNBC and SCLC (60).

Moreover Deng et al. (2021) shows that ICIs plus platinum-free, single-agent CT can provide promising progression-free survival and overall response rate benefit, along with a low rate of severe adverse events in patients with epidermal growth factor receptor-tyrosine kinase (EGFR-TKI)-resistant advanced NSCLC (61). The result of another research shows that triple combination therapy with CT or agents eliciting oncolytic virus-like responses may overcome multiple resistance mechanisms (62).

## Immunologic aspects of radiation therapy

RT has been widely used in oncology practice as a monotherapy or in combinations with other therapeutic strategies depending on the disease and patient characteristics (63). The first experimental observation pointing to the role of the immune system in antitumor activity of RT was obtained in 1979: the radiation dose required to control the tumor in 50% of mice was twice as large in the group of immunodeficient animals compared to the control group (64). Immune-mediated indirect suppression of tumor metastases by RT was first reported by R. Mole et al. in 1953 and called «abscopal effect» (65). Many years later, in 2004 S. Demaria et al. demonstrated importance of the immune system in abscopal effect of RT implying distant control of unirradiated tumor lesions in mouse (66). In summary, mechanism of RT action is complex and includes both direct cytotoxic effect of RT on cancer cells and an immune-related component (67).

**Figure 2:**
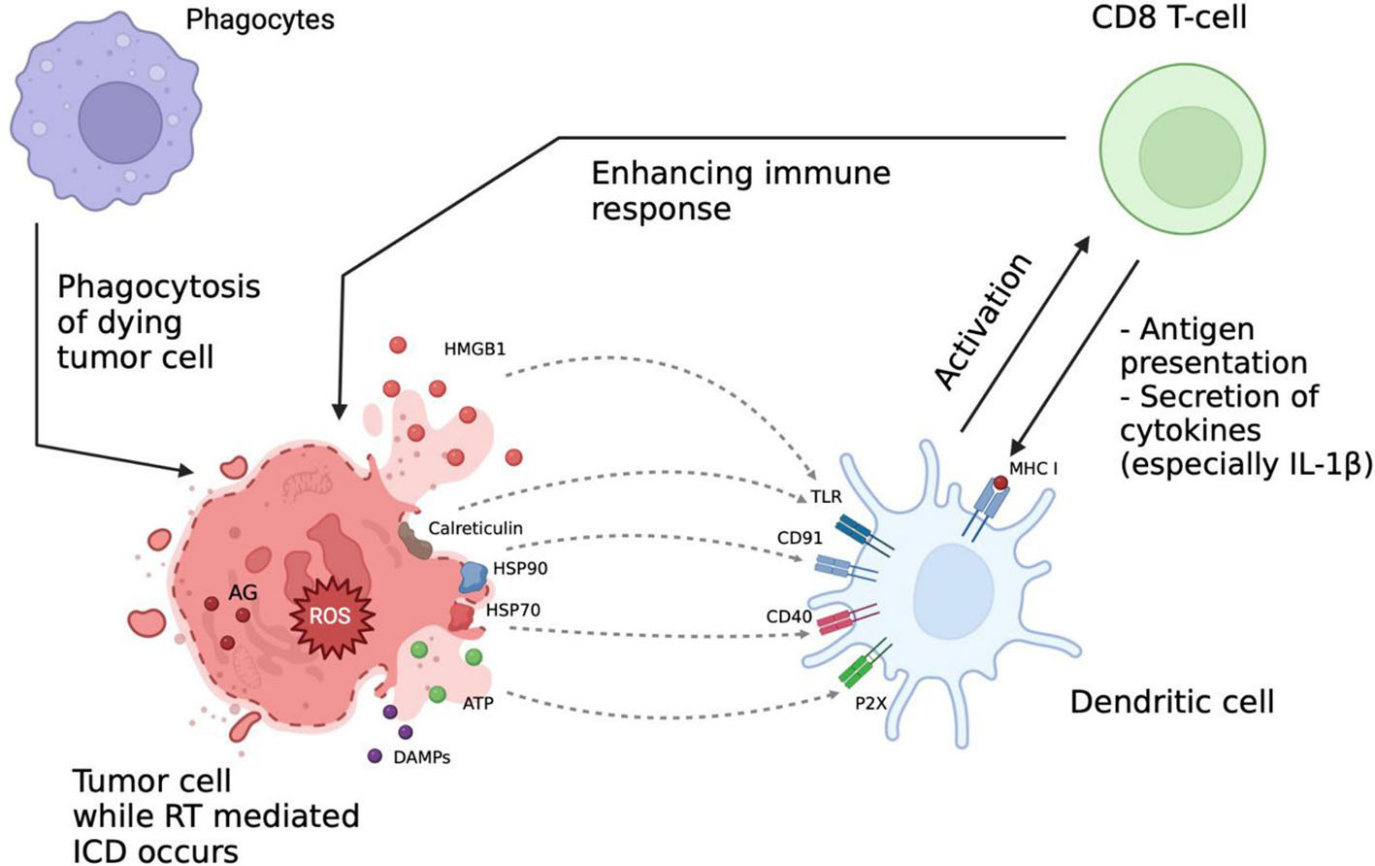
Immunologic aspects of radiation therapy (RT). RT effect is mediated *via* formation of reactive oxygen species (ROS) affecting the DNA structure and leading to immunogenic cell death (ICD). This process is accompanied by release of tumor antigens (AG) and damage-associated molecular patterns (DAMPs) into extracellular space and activation of signaling pathways involved into the immune response. After RT the interaction between immune cells is increased, ICI can further enhance it, thus increasing the overall therapeutic efficacy. AG, tumor-associated antigens; DAMPs, damage associated molecular patterns; ICD, immunogenic cell death; ROS, reactive oxygen species; HMGB1, high-mobility group protein B1; HSP70, heat shock protein 70; HSP90, heat shock protein 90; TLR, toll-like receptors; MHC I, major histocompatibility complex class I.

Recent studies showed that the therapeutic effect of RT is mediated by formation of reactive oxygen species (ROS) affecting DNA structure and leading to ICD. This process is accompanied by multiple changes in molecular signaling pathways and intercellular interactions such as release of tumor antigens and damage-associated molecular patterns (DAMPs) into extracellular space followed by activation of signaling pathways involved in the immune response (**Figure 2**) (23, 68–70).

The RT-induced immune response is facilitated by release of adenosine triphosphate (ATP), uric acid and other intracellular components serving as chemo-attractants for APC (69). ATP, released in millimolar concentrations, promotes phenotypic maturation of the DCs by binding to their purinergic P2X and P2Y receptors (71–73). Interestingly, upon activation of the P2X7 receptor DCs can synthesize the precursor IL-1β, which is an essential component of the antitumor immune response (74, 75).

Secretion of pro-interleukin is carried out by a cascade activation mechanism including the nod-like receptor family pyrin domain containing 3 (NLRP3) and inflammasome-mediated secretion with following caspase-1-mediated processing (76, 77). It is important to note that the secretion of IL-1β requires concomitant signaling from the P2X7 receptors and toll-like receptors (TLRs), which can be suppressed by heat shock proteins (HSPs) and calreticulin (78, 79). Uric acid can additionally promote the immunological component by stimulating nucleotide-binding oligomerization domain-like receptor NALP3 *via* urate ion interactions with TLR4 (80, 81).

Alternatively, to the mentioned above ATP-dependent mechanisms of immune response, Y. He et al. (2013) demonstrated stimulation of cytokine secretion without high interstitial concentrations of ATP in a mouse model, suggesting a role of autocrine DCs activation (82).

As a protective reaction to irradiation tumor cells increase surface expression of various plasma membrane proteins, including HSPs and high-mobility group box 1 (HMGB1) (83). HSPs play a critical role in enhancing immunogenicity by signaling to APC and stimulating phagocytosis and cross-presentation of antigens mediated by NK cells. Specifically, HSPs 70 and 90 can bind to CD40 and CD91 receptors of DCs (84). Moreover, HSP70 has been shown to activate the cytotoxic effect of CD8 T cells after interacting with the co-activating molecule CD40 (85), and the interaction of HSP90 with CD91 potentiates the killing of tumor cells *via* cross-presentation of tumor antigens by DCs (86).

In this context, irradiation causes formation of necrotic tumor areas, characterized by calreticulin overexpression at the cell surface (87), which stimulates phagocytosis of tumor cells and further antigen release promoting the immune response (88, 89). Numerous studies show that it is calreticulin that plays the vital role in potentiating the immune system activity, since the level of its expression on the plasma membrane surface is directly proportional to the degree of immunocompetent cells attraction to the inflammation focus and antigen-specific T cell response (89). In addition, various studies have demonstrated a correlation between calreticulin expression and overall patient survival, which clearly distinguishes calreticulin from other DAMPs suggesting potential use of calreticulin as a prognostic marker for success of the RT in various types of cancer (89–92).

Additional evidence for stimulation of the antitumor immune response caused by radiation comes from the expression of HMGB1 (a nuclear DNA-binding protein synthesized during cell death) on the surface of tumor cells, which promotes its processing by DCs *via* TLR-mediated pathways and the receptor of advanced glycation end-products (RAGE)-mediated signaling (93, 94).

Moreover, irradiation increases MHC class I expression on the surface of cancer cells, which allows cancer-specific T cells to recognize and destroy the tumor cells. Increased expression of MHC class I is one of the best-characterized major mechanisms for enhancing immune responses to radiation. The activation of mammalian target of rapamycin (mTOR), enhanced translation and antigen presentation are crucial for this process. Enhanced surface expression of Fas induced by radiation promotes the apoptotic tumor cells death and represents another important mechanism mediating effects of RT on the host immune system (95).

The T cell immune response has been shown to be proportionally stimulated by the released of tumor antigen according to the radiation dose (96, 97). The RT destroys the tumor cells leading to the local activation of immune system with later generalization of the anti-tumor immune response. DAMPs may induce migration of neutrophils in the tumor site and enhance antitumor immune response as well (98). Attracted by sterile inflammation to the area of irradiation, neutrophils destroy tumor cells with free oxygen radicals and ensuing phagocytosis (98).

Thus, irradiation contributes to tumor necrosis, inflammation in the tumor site and multiple responses of the immune system to the tumor (99). Among various forms of cell death caused by ionizing radiation, ICD is caused by direct stimulation of tumor-specific immune response. Apart from activating anti-tumor immunity, RT may cause immunosuppressive effects as well. In this context, increased expression of PD-1 and its ligand PD-L1 in tumor in tumor microenvironment may serve as an important predictor for ICI administration (100, 101). Combining RT with ICI may also help to overcome local immunosuppressive effects originating from tumor cells.

**Figure 3:**
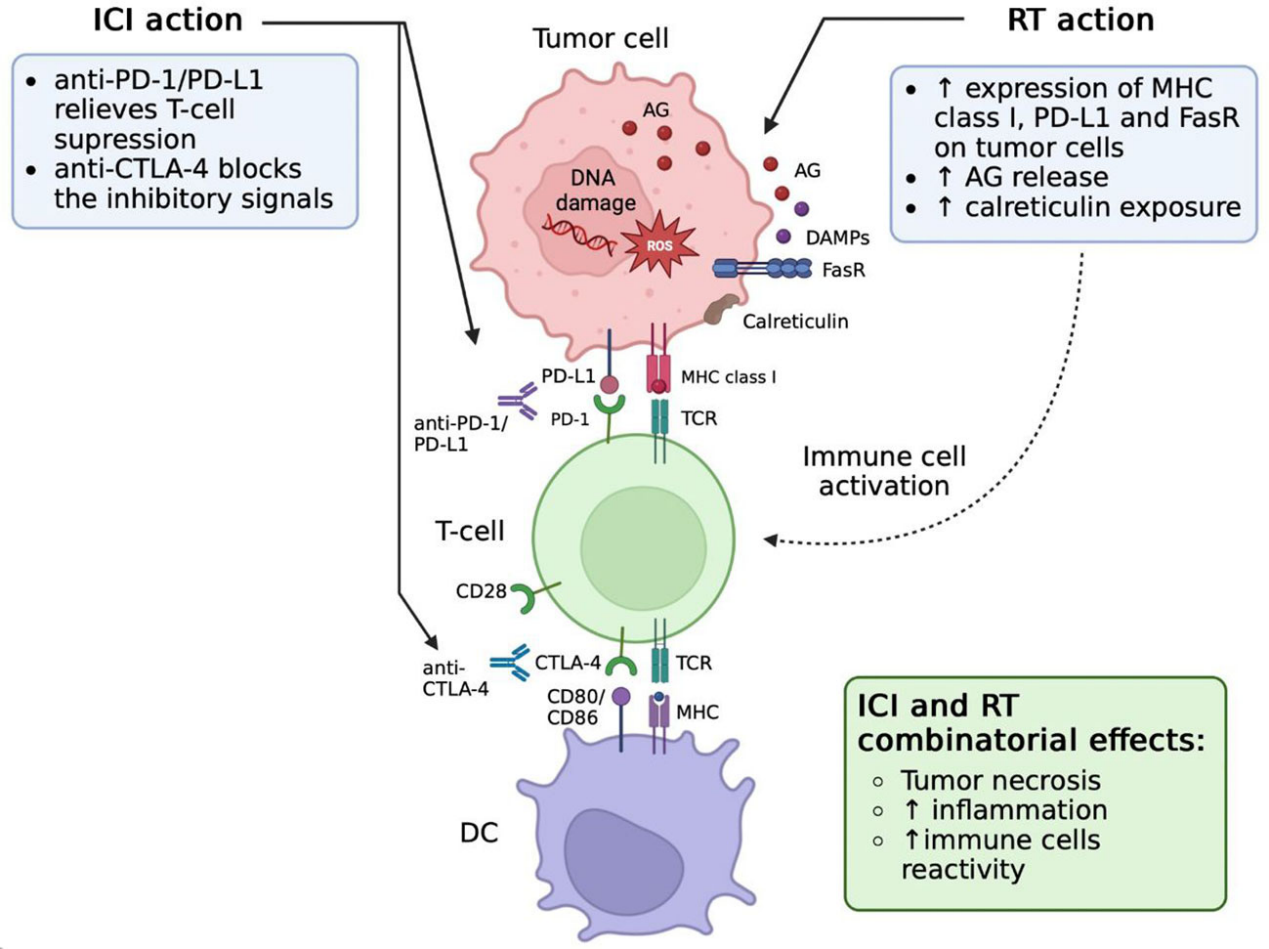
Effects of radiation therapy (RT) and immune checkpoint inhibitors (ICIs) combination. RT induces PD-L1 upregulation on tumor cells. Blockade of PD-L1/PD-1 signaling *via* antibody therapy repairs the function of CD8 T cells after RT stimulation. These functionally active CD8 T cells are able to effectively attack and kill cancer cells, which leads to tumor cell necrosis and inflammation increase. AG, tumor-associated antigens; CTLA-4, cytotoxic T-lymphocyte-associated protein 4; DAMPs, damage-associated molecular patterns; DC, dendritic cells; ICI, immune checkpoint inhibitors; MHC, major histocompatibility complex; PD-1, programmed cell death protein 1; RT, radiotherapy; TCR, T-cell receptor; FasR, Fas receptor.

The current state of knowledge strongly suggests that RT acts not only on the irradiated tumor site but also elicits a specific immunologic response, which puts forward the combination of radio- and immunotherapy as a promising strategy for improved management of metastatic tumor conditions. The systemic effect of RT on the immune system is further manifested by enhancing the homing effect of NK cells, that is an essential factor of innate antitumor immunity (102). In addition, RT can decrease the suppressive effect of Treg cells which usually down modulate immune responses against cancers (103). At the same time, irradiation has been associated with immunosuppressive mechanisms such as PD-L1 expression (21, 104). Therefore, therapeutic blockade of the PD-1/PD-L1 axis may be considered as a strategy to enhance the antitumor effect of RT (**Figure 3**). However, these results were obtained in animal models and need further verification.

The RT exerts not only a direct effect in the area of irradiation but also indirectly stimulates the immune response due to low doses of irradiation beyond the target zone. These low irradiation doses may stimulate an immune response to the general stress, caused by radiation, including the adaptive response and immune system repair (103). The reduction of immune tolerance to tumors caused by RT may amplify the general anti-tumor immune response and mediate the abscopal effect, although mechanisms underlying the abscopal effect require further characterization.

## Preclinical efficacy of radio- and immunotherapies

Summarizing the available data, it can be stated that a combination of RT and immunotherapies may represent a biologically rationale approach to improve cancer treatment outcomes (19). Synergy of these treatment modalities has been extensively investigated in several mouse models of cancer (105).

As was mentioned earlier, rational selection of the radiation dose, fractional content and the sequence of prescribing the therapy components are required to achieve the most pronounced treatment benefits. Fractionation (separation of the total dose of radiation into several fraction) allows maximum destruction of malignant cells with minimal damage to healthy tissues.

Currently, there are three optimal modes of dose fractionation used in RT:

- Hypofractionation (3-20 Gy/fraction, one fraction/day, 2-5 fractions/week);
- Conventional fractionation schemes (1.8-2.2 Gy/fraction, one fraction/day, 5 days/week for 3-7 weeks);
- Hyperfractionation (0.5-2.2 Gy/fraction, two fractions/day, 2-5 fractions/week for 2-4 weeks) (106).

Immunological effects of different regimens and the molecular mechanisms underlying the immune response to radiation remain unclear. It’s established that single high-dose (12 Gy) RT did not deplete CD8 T cells but kills tumor cells more effectively when combined with immunotherapy (107).

In experimental mouse models after exposure to hypofractionated RT a strong upregulation of PD-L1 was observed (radiation and anti-PD-L1 treatment synergistically promote antitumor immunity in mice), so we can suppose that the combination of the PD-1/PD-L1 blockade and RT may overcome tumor immunosuppression and improve the systemic effect of RT.

In contrast to the conventional and hypofractionated regimen, the single, high-dose irradiation did not increase the surface expression of PD-L1 on B16-F10 melanoma and GL261 glioblastoma cells *in vitro* (22). The positive effect of a single exposure has been also established in other studies. It was demonstrated that a single high dose of radiation (12 Gy) does not cause the death of immunocompetent cells suggesting that combination of this regimen with ICI may be successful (107). Therapeutic potential of combining PD-1 blockade with a single exposure of 10 Gy has been further demonstrated in a mouse model of glioma (108).

Other studies demonstrated the superiority of fractionated RT over single-session RT. Fractionated RT appears to be more synergistic with ICI therapy, inducing tumor regression and increasing long-term survival rate in various extracranial cancer models (109). It was suggested that the single dose RT has a positive effect on the micrometastases regression but is not active enough against the mature ones. Comparison of two dose regimen of fractionated therapy (3*8 Gy and 5*6 Gy) in combination with CTLA-4 blockade showed that the dose of 3*8 Gy was more efficient (109).

There is evidence for the role of the size of the ablative radiation fraction (minimum 6 Gy), as well as the linear energy transfer level affect the release of immunogenic antigens. A. Lugade et al. (2005) compared 15 Gy in one fraction versus 15 Gy in 5 fractions of 3 Gy in a murine melanoma model. They found that fractions equally lead to tumor infiltration by lymphocytes, but larger fraction sizes produce a better effect (110).

The DNA exonuclease three prime repair exonuclease 1 (TREX 1) may serve as a marker for the optimal dose determination, since its expression proportionally correlates with the radiation dose and reflects the severity of the radiation-induced DNA-damage (111). Cells treated with a single dose of 20 and 30 Gy showed a larger increase in TREX1 exonuclease expression than cells treated with 3*8 Gy. High dose radiation (above 12-18 Gy) in a single fraction promotes the TREX 1-induced DNA degradation and cytosolic accumulation of damaged DNA in irradiated cancer cells, which inhibits the type-I interferon (IFN-I) pathway and decreases the immunogenicity of cancer cells. In contrast, the radiation dose of 3*8 Gy given in a repeated fashion is below the threshold dose for TREX 1 induction. This dose regimen is also optimal in terms of the IFNβ stimulation required for recruitment of Batf3-dependent DCs and CD8 T-cells to the tumor site. Use of ICI, in this context, may promote a sustained regression of the irradiated and non-irradiated tumors *via* direct and abscopal effects.

Notably, the success of combination therapy is influenced not only by the fractionation scheme but also depends on the type of immunotherapeutic intervention, as well as sequence of the immunotherapeutic and RT modalities. Concurrent PD-1/PD-L1 blockade and RT was shown to be more effective compared to sequential administration of RT followed by ICI (21), whereas anti-CTLA-4 therapy was shown to be the most effective when given before RT in a colorectal cancer model (112). Therefore, disease-specific approaches and personalized medicine should be applied for decisions on the concrete strategy.

Thus, optimal modes of radiation and immunotherapy in pre-clinical studies are various and are determined primarily by the mechanics of action of a specific immunotherapy agent. This should be taken into account when designing clinical trials.

## Clinical usage of radio- and immunotherapies

It should be stated that the first clinical trials of combined radio- and immunotherapy were conducted in 80th and included administration of cancer vaccines and RT for treatment of melanoma, breast, colorectal or lung cancer but failed to demonstrate the efficacy of combined therapy (113). Following decades of oblivion, the interest to the radio- and immunotherapies was renewed after ICI discovery.

Clinical benefit of combining ICI and RT was initially described by multiple retrospective analyses comparing efficacy of RT alone or RT in combination with ICI in patients with highly immunogenic cancer forms such as metastatic melanoma, NSCLC and RCC (114). The following prospective clinical trials have corroborated the therapeutic potential of the combined ICI and RT approach (**Table 2**) (119, 120). Identification of the optimal sequencing strategy has been performed retrospectively and comprehensive meta-analysis of the accumulated clinical evidence suggested superiority of concurrent over sequential ICI and RT treatment (121). In contrast to the retrospective observations, the prospective single institution ELEKTRA trial demonstrated superiority of RT given prior to ICI treatment, which was reflected by more pronounced increases in circulating CD4 and CD8 cell populations (122). These discrepancies may be related with confounding factors, affecting conclusions of the retrospective trials (122).

**Table 2:** Examples of clinical trials evaluating efficacy of RT+ICI combinations.

Indication	Phase	Clinical outcomes		Reference
**Melanoma brain metastases**	II	SRS and Ipilimumab (n = 45)	6-month intracranial PFS 48% 12-month intracranial PFS 17% 12-month extracranial PFS 17% OS 68%	(4)
		SRS and Nivolumab (n = 35)	6-month intracranial PFS 69% 12-month intracranial PFS 42% 12-month extracranial PFS 37% OS 78%	
**Diffuse intrinsic pontine glioma**	R	reRT and Nivolumab (n=8)	OS 22.9 months (p<0.0001)	(115)
		reRT alone (n=4)	OS 20.4 months (p<0.0001)	
		No reRT or ICI (n=19)	OS 8.3 months (p<0.0001)	
**Advanced solid tumors**	I	SBRT and Pembrolizumab (n=79)	OR 13.2% OS 9.6 months PFS 3.1 months	(116)
**Metastatic non-small cell lung cancer**	III			(117)
		CT and RT and Durvalumab (n = 473)	PFS 16.8 months	
**Advanced melanoma**	III	Ipilimumab and RT (n=70)	OS 19 months (p=0.01) PFS 5 months (p=0.20) CR 25.7% (p=0.04) OR 37.1% (p=0.11)	(118)
		Ipilimumab alone (n=31)	OS 10 months (p=0.01) PFS 3 months (p=0.20) CR 6.5% (p=0.04) OR 19.4% (p=0.11)	

SRS, stereotactic radiosurgery; SBRT, stereotactic body RT; OS, overall survival; PFS, progression free survival; CR, complete response; OR, overall response; R, retrospective trial.

Efficacy of the ICI and RT combination has been also investigated in other tumor types. A retrospective analysis of diffuse intrinsic pontine glioma patients treated with RT alone or RT in combination with PD-1 specific antibodies demonstrated no additional benefit of ICI inclusion into the therapy. Two prospective trials Checkmate-498 and Checkmate-548, evaluating efficacy of the triple combination of nivolumab with RT and temozolomide in patients with primary glioblastoma have failed to meet primary endpoints. The lack of the combination efficacy may be related to low activity of immunotherapies by this indication due to low tumor immunogenicity as well as activation of alternative immunosuppressive pathways (115, 123). In contrast, another prospective study Checkmate-577 demonstrated improvement of the esophageal or gastroesophageal Junction cancer treatment outcomes following the addition of nivolumab to neoadjuvant chemoradiotherapy (124). Efficacy of ICI and RT combination has been also tested in other indications such as metastatic breast (NCT03483012, NCT03807765), pancreatic (NCT0436116), ovarian cancers (NCT03283943), hematological malignancies (NCT03610061) and hepatocellular carcinoma (NCT04913480).

Therefore, the effectiveness of combined radioimmunotherapy depends on the choice of the optimal radiotherapy mode, including both the total dose and the fractionation mode.

The different contribution of TME components of a particular patient to the regulation of the immune system, depending on the stage of immune response development, determines the significance of the RT sequence and the taking of immune drugs.

Prospective predicting of clinical responses to combined radioimmunotherapy is possible by identifying predictive biomarkers. Currently two biomarkers (PD-L1 and MMR/MSI status) have already been implemented in the clinic. Soluble NKG2D ligands and antibodies that neutralize their activity are considered to be easily accessible predictive biomarkers for patients receiving combination of RT and ICIs (125, 126). RT promotes the exposure of NKG2D ligands on the surface of tumor cells, hence rendering them potentially susceptible to NK cell-dependent lysis or improved recognition by CTLs (125). Cancer cells can shed ligands from their surface, resulting in high circulating levels and can have a negative predictive value in melanoma patients treated with various ICIs including nivolumab, pembrolizumab and ipilimumab (127–131).

Markers of cGAS-STING DNA-sensing pathway which is essential for activation of IFN-dependent antitumor immunity are also promising for the identification of patients with positive response to combinatorial regimens involving RT and ICIs (132).

## Conclusion

Therapeutic benefits of combined ICI and RT approach has been suggested by numerous studies enrolling patients with melanoma, NSCLC and RCC and is currently being investigated in other indications. The optimal therapeutic regimen in terms of the doses, RT fractionation and sequence of RT and ICI administration has been addressed in preclinical setting but needs a further corroboration in clinical trials. The available studies categorize the combination of ICI and RT as a promising approach for improved treatment of immunogenic cancer forms.

## Author contributions

Conceptualization: KP, VV, SL, MS. Literature review: MM, VV, AV. Writing: MM, MSa, AV. Editing: KM, VV, MS. Visualization: MM, NB, SL. Supervision: SL. All authors contributed to the article and approved the submitted version.

## Funding

This work was financed by the Ministry of Science and Higher Education of the Russian Federation within the framework of state support for the creation and development of World-Class Research Centers “Digital biodesign and personalized healthcare” №075-15-2022-304

## Conflict of interest

Authors VV and KP were employed by company M&S Decisions LLC.

The remaining authors declare that the research was conducted in the absence of any commercial or financial relationships that could be construed as a potential conflict of interest.

## Publisher’s note

All claims expressed in this article are solely those of the authors and do not necessarily represent those of their affiliated organizations, or those of the publisher, the editors and the reviewers. Any product that may be evaluated in this article, or claim that may be made by its manufacturer, is not guaranteed or endorsed by the publisher.
